# LXRα promotes cell metastasis by regulating the NLRP3 inflammasome in renal cell carcinoma

**DOI:** 10.1038/s41419-019-1345-3

**Published:** 2019-02-15

**Authors:** KeShan Wang, TianBo Xu, HaiLong Ruan, HaiBing Xiao, Jingchong Liu, ZhengShuai Song, Qi Cao, Lin Bao, Di Liu, Cheng Wang, Gong Cheng, HuaGeng Liang, ZhaoHui Chen, HongMei Yang, Ke Chen, XiaoPing Zhang

**Affiliations:** 1 Department of Urology, Union Hospital, Tongji Medical College, Huazhong University of Science and Technology, Wuhan, 430022 China; 2 Institute of Urology, Union Hospital, Tongji Medical College, Huazhong University of Science and Technology, Wuhan, 430022 China; 3 Department of Pathogenic Biology, School of Basic Medicine, Huazhong University of Science and Technology, Wuhan, 430030 China

## Abstract

Notwithstanding the researches on biomarkers and targeted therapies in renal cell carcinomas (RCC) have made progress in the last decades, the application of the biomarkers and targeted therapy agents for RCC in clinic are restricted because of their limitation or side effects. Liver X receptors (LXRs) and the NLRP3 inflammasome have been the research hotspots in recent years. In our study, we integrated bioinformatics analysis, molecular biology experiments and biological function experiments to study the roles of LXRα and the NLRP3 inflammasome in RCC. The study demonstrated that the elevated LXRα expression is correlated with a poor prognosis in RCC. Furthermore, our study revealed the expression levels and roles of the NLRP3 inflammasome in RCC for the first time. This research demonstrated that LXRα could promote the metastasis of RCC cells by suppressing the expression of the NLRP3 inflammasome. In Brief, LXRα had the possibility to be a novel diagnostic and prognostic biomarker and therapeutic target in renal cell cancer and LXRα could regulate the metastasis of renal cell cancer via NLRP3 inflammamsome.

## Introduction

Renal cell carcinoma (RCC), characterized by high incidence and high mortality rates, constitutes 3% of adult malignancies. It is estimated that 65,340 new cases of RCC will be diagnosed and that 14,970 patients will die from RCC in 2018 in the USA.^1^. Clear-cell renal cell carcinoma (ccRCC) accounts for 75% of renal cell carcinomas and is the most familiar subtype of RCC^2^. According to reports, 20–30% RCC patients present with local or distant metastasis at the time of diagnosis, and the response to chemotherapy or radiotherapy in the majority of the advanced RCC patients is dissatisfying^3^. We considered that the exploration and application of effective diagnostic and prognostic biomarkers for RCC patients could be useful for ccRCC diagnosis and therapy. Although the occurrence of target drugs for RCC have improved therapeutic outcomes of advanced and metastatic RCC, poor response and high incidence of side effects for these agents restrict their clinical application^4^. Therefore, it is urgent to explore new potential biomarkers and therapeutic targets for RCC.

As a kind of nuclear receptor superfamily, the liver X receptor (LXR) family, including LXRα (LXRA, NR1H3) and LXRβ (LXRB, NR1H2), is an important regulator of several kinds of cancers (prostate cancer, breast cancer, etc.). While LXRβ is expressed widely throughout various tissues and organs, LXRα is mainly expressed in liver, kidney, spleen and intestine^5–8^. According to the studies, LXRs could influence the inflammatory response via regulating the expression of inflammatory cytokines, including STAT, IL-18, TNF-α and so on^9–11^. The inflammasome, a novel research focus, is a kind of cytosolic multi-protein complex which could participate in the development of several cancers via regulation of tumor inflammation and immunity^12,13^. The NLRP3 inflammasome, one of the most critical inflammasomes, contains NLRP3, apoptosis-associated speck-like protein containing a CARD (ASC), and caspase-1 (CASP1). The NLRP3 inflammasome functioned as a platform for caspase-1 activation and the activated caspase-1 could cleave the pro-IL-1β into mature IL-1β^14^. According to the researches, the NLRP3 inflammasome could regulate the development of the cancers. Activating of the NLRP3 inflammasome was correlated with the azoxymethane-induced colorectal cancer^15^. In lung cancer, the NLRP3 inflammasome could enhance the cell proliferation and migration and was a potential therapy target for lung cancer^16^. Knockdown of NLRP3 could inhibit the proliferation and invasion of pancreatic cancer cells^17^. Activation of the NLRP3 inflammasome could promote the carcinogenesis in squamous cell carcinoma of the head and neck^18^.

The expression and biological functions of LXRs and the NLRP3 inflammasome, as well as their possible correlation to RCC, is still unclear. Based on the above studies, firstly, we explored the expression level and function of LXRα in RCC. Our study indicated that LXRα could be a biomarker for diagnosis and prognosis in ccRCC. Moreover, we investigated the mechanism of LXRα regulating the development of RCC and the study revealed that LXRα could regulate the metastasis of ccRCC via the NLRP3 inflammasome for the first time.

## Results

### Elevated LXRα expression correlates with clinical features in ccRCC

**Fig. 1:**
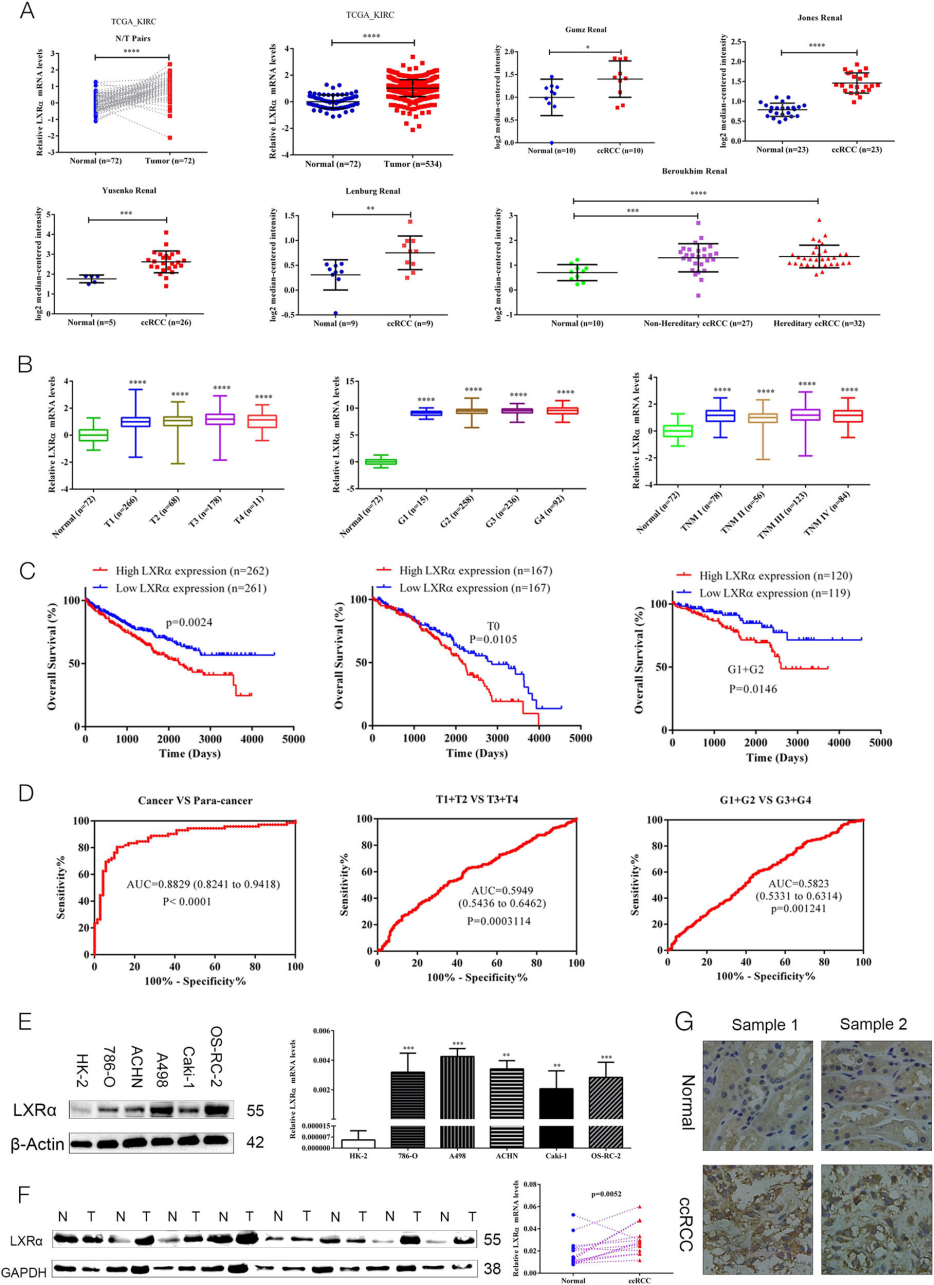
LXRα expression is elevated in ccRCC and correlates with clinical features. **a** mRNA levels for LXRα were obtained from TCGA_KIRC datasets and Oncomine datasets (Gumz Renal, Jones Renal, Yusenko Renal, Lenburg Renal, and Beroukhim Renal). **b** mRNA levels of LXRα compared with different clinicopathological features: T stage, G stage and TNM stage. **c** The OS of ccRCC patients is correlated with expression of LXRα, and high expression of LXRα indicates a poor prognosis in T0 and G1+G2 stage ccRCC patients. **d** Expression of LXRα differentiates the adjacent normal tissues and ccRCC cancer tissues according to the ROC curve analysis. ROC curve analysis of LXRα expression in subgroups of ccRCC patients with (T1+T2) VS (T3+T4) stage and (G1+G2) VS (G3+G4) stage was also performed. **e** Western blotting and qRT-PCR analysis of LXRα protein and mRNA expression levels in ccRCC cell lines and HK-2 cells. **f** Expression levels of LXRα protein and mRNA in ccRCC cancer tissue and normal kidney tissue. **g** Immunohistochemistry (IHC) analysis of LXRα expression in ccRCC tissues and normal kidney tissues. Original magnification x200 (Data are shown as mean ± SD. ****P < 0.0001; ***P < 0.001; **P < 0.01; *P < 0.05, and ns means no significant difference compared with the corresponding control)

Although bioinformatics analysis of LXR (LXRα and LXRβ) expression in ccRCC indicated that both LXRα and LXRβ mRNA expression in ccRCC cancer tissue was higher than that in corresponding adjacent normal tissues, the elevated degree of LXRα expression in ccRCC cancer tissues compared with normal kidney were much higher than those of LXRβ (Fig. 1a, Fig. S1A). Further bioinformatics analysis of LXRα in ccRCC indicated that its expression levels were related with clinicopathological features in ccRCC patients (Table 1, Fig. 1b). Kaplan-Meier curves indicated that high expression of LXRα or LXRβ predicted a poor prognosis in ccRCC patients, and the overexpression of LXRα in ccRCC patients who were T1+T2 stage (*p* = 0.0105) or G1+G2 stage (*p* = 0.0146) was also associated with a poor prognosis (Fig. 1c, Fig. S1B). Additionally, LXRα effectively differentiated ccRCC from paracancerous normal tissues with an area under the curve (AUC) of 0.8829 (95% CI: 0.8241–0.9418; *p* < 0.0001), and LXRα expression may be a potential diagnostic biomarker for ccRCC patients with (T1+T2) VS (T3+T4) stage and (G1+G2) VS (G3+G4) stage according to the results of ROC curves (Fig. 1d). To verify LXRα expression levels in ccRCC cell lines and tissues, we extracted the RNA and protein from both ccRCC cells and tissues for qRT-PCR assay and western blotting analysis, respectively. Our results indicated that the protein and mRNA expressions of LXRα were up-regulated in both ccRCC cells and tissues (Figs. 1e, f). Simultaneously, results of IHC analysis of LXRα also revealed the same result (Fig. 1g). In addition, the qRT-PCR analysis of LXRβ demonstrated that the degree of elevations of LXRβ expression in ccRCC cancer tissues were lower than LXRα expression (Fig. S1C). These findings revealed that the expression of LXRα is elevated in ccRCC cancer tissues and that the expression of LXRα was correlated with the clinical features of ccRCC and could be a potential biomarker for ccRCC diagnosis and prognosis.

**Table 1:** Correlation between LXRα mRNA expression and clinicopathological parameters of KIRC

Parameter	Number	LXRα mRNA expression		*P* value
		Low (*n* = 261)	High (*n* = 262)	
Age (years)				
<60	260	144	116	**0.013**
>=60	263	117	146	
T stage				
T1 + T2	334	187	147	**0.000**
T3 + T4	189	74	115	
N stage				
N0	507	254	253	0.617
N1	16	7	9	
M stage				
M0	445	229	216	0.089
M1	78	32	46	
Neoplasm histologic grade				
G1 + G2	244	140	104	**0.001**
G3 + G4	279	121	158	
Pathologic stage				
1 + 2	316	181	135	**0.000**
3 + 4	207	80	127	
Gender				
Male	341	157	184	**0.016**
Female	182	104	78	
Laterality				
Left	246	116	130	0.236
Right	277	145	132	

Significance of bold values are *p* < 0.05

### LXRα regulates cell migration and invasion in ccRCC cells

**Fig. 2:**
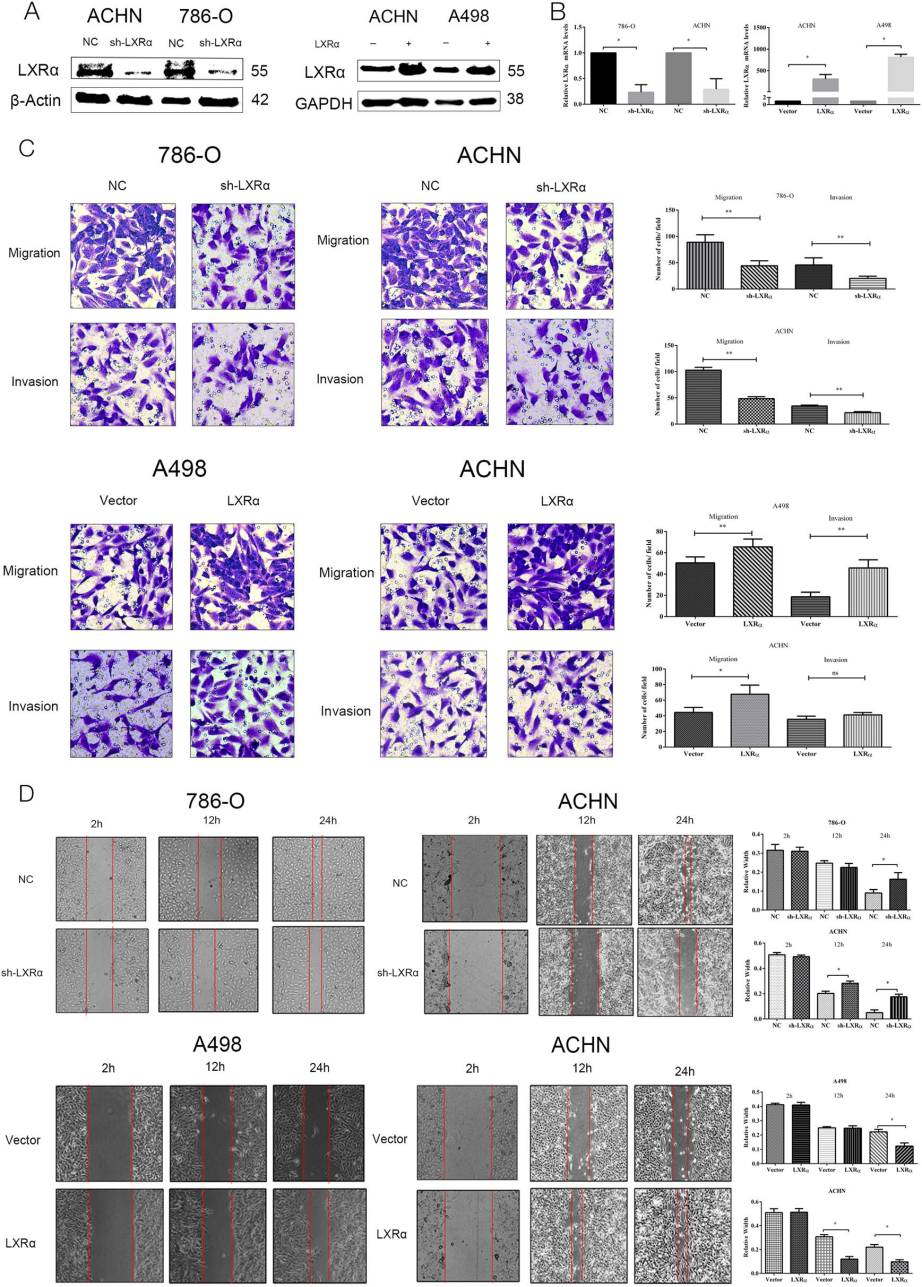
LXRα expression level is correlated with ccRCC cell migration and invasion. Results of stable infection of sh-LXRα and LXRα in ccRCC cells are shown for protein level (**a**) and mRNA level (**b**) of LXRα. (**c**, **d**) Transwell migration and invasion and wound healing assays of ccRCC cells with stable transfection of sh-LXRα versus control (NC) (left two columns) or LXRα versus Vector (right two columns) were performed. The wound healing assays were imaged at 2, 12, and 24 h after scratches were made. Original magnification x200 and x100 (Data are shown as mean ± SD. ****P < 0.0001; ***P < 0.001; **P < 0.01; *P < 0.05, and ns means no significant difference compared with the corresponding control)

According to the results above, we predicted that both LXRα and LXRβ might be oncogenes in ccRCC. However, elevation degree of LXRα in ccRCC cancer tissue compared to normal kidney tissues was much higher than that of LXRβ. Furthermore, reports have also revealed that expression of LXRα is mainly in lipid metabolism-related tissues (kidney, liver, etc.), while LXRβ is widely expressed across tissues. Thus, we considered that LXRα expression in renal cancer may be more specific and functional than LXRβ and we decided to focus on the roles of LXRα in ccRCC in our study. To verify the function of LXRα in ccRCC, we utilized ACHN and 786-O cells with stable knockdown of LXRα and ACHN and A498 cells with stable overexpression of LXRα. Cell transfection efficiency and induction of constructs were verified via western blotting and qRT-PCR (Figs. 2a, b). Furthermore, our results indicated that expression of LXRα in ccRCC was mainly correlated with aggressiveness in ccRCC. In transwell assays, 786-O and ACHN normal control cells possessed greater migration and invasion capacity than corresponding LXRα stable knockdown cells. Congruously, cell migration and invasion in A498 and ACHN cells with stable overexpression of LXRα were faster compared to corresponding normal control cell lines (Fig. 2c). Similarly, wound healing assays revealed that the knockdown of LXRα could suppress the scratch healing capacity of ccRCC, while the overexpression of LXRα could enhance healing ability (Fig. 2d). In addition to transwell assays, apoptosis and cell cycle assays revealed that knockdown of LXRα had almost no effect on these processes in ccRCC cells (Fig. S2A, B). Collectively, these results demonstrated that LXRα expression mostly enhanced the cell aggressiveness ability of ccRCC cells.

### LXRα could negative regulate the expression level of the NLRP3 inflammasome in ccRCC cells

**Fig. 3:**
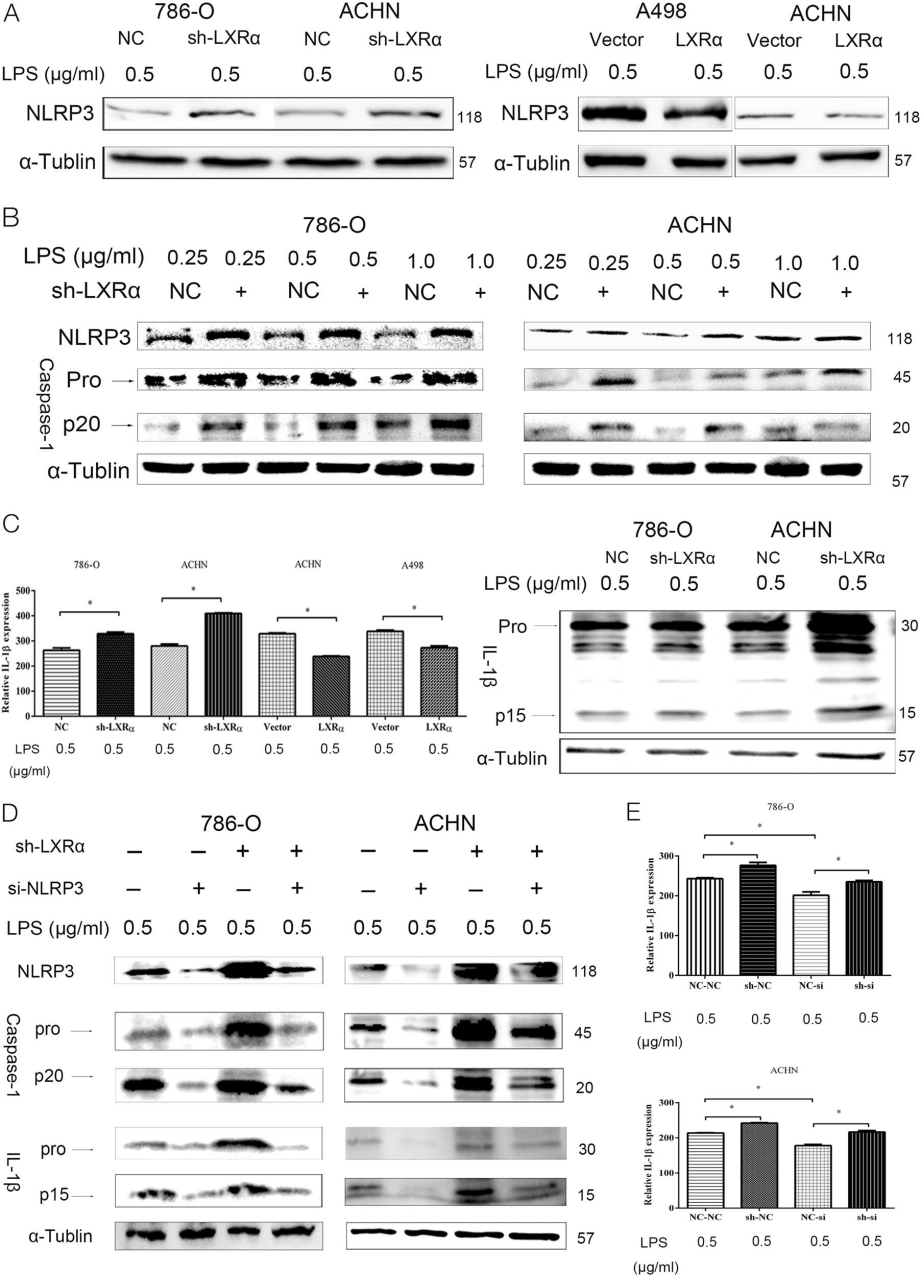
NLRP3 inflammasome expression is negatively regulated by LXRα in ccRCC cells. **a** Expression levels of NLRP3 were elevated in 786-O and ACHN cells with stable LXRα knockdown. NLRP3 expression was down-regulated in A498 and ACHN cells with LXRα overexpression. **b** Expression levels of NLRP3, pro-CASP1, and cleaved CASP1 (p20) in 786-O and ACHN cells with stable LXRα knockdown. LPS stimulus was performed at 0.25, 0.5, and 1.0 µg/ml. **c** ELISA assays were used to analyze exocrine IL-1β expression levels in culture supernatant from ccRCC cells with stable LXRα knockdown or LXRα overexpression. Protein expression of pro-IL-1β and cleaved IL-1β (p15) in 786-O and ACHN cells with stable LXRα knockdown were detected by western blotting. **d** Transient transfection of si-NLRP3 inhibited induction of pro-CASP1, cleaved CASP1 (p20), pro-IL-1β and cleaved IL-1β protein expression, which were elevated in stable LXRα knockdown cells. **e** Elevation of exocrine IL-1β expression in 786-O and ACHN cells with stable LXRα knockdown was blocked by transient transfection with si-NLRP3. NC-NC, sh-NC, NC-si, and sh-si indicates NC+NC, sh-LXRα+NC, NC+si-NLRP3, and sh-LXRα+si-NLRP3, respectively. (Data are shown as mean ± SD. ****P < 0.0001; ***P < 0.001; **P < 0.01; *P < 0.05, and ns means no significant difference compared with the corresponding control)

After we confirmed the biological function of LXRα in ccRCC, we explored the possible mechanisms enhancing tumor growth, migration and invasion of LXRα in ccRCC cells. As we mentioned before, LXRα could regulate the expression of typical inflammatory cytokines. However, the relationship between LXRα, the NLRP3 inflammasome, and the biological effects of their correlation in ccRCC have not been reported. To investigate these problems, we evaluated the expression of NLRP3 in ccRCC cancer tissues. Our study revealed that the expressions of NLRP3 in ccRCC cancer tissues were significant lower than that in normal kidney tissues for the first time (Fig. S2C). According to the western blotting results, knockdown of LXRα could up-regulate the expression of NLRP3 in ccRCC cells, while LXRα overexpression could decrease the expression level of NLRP3 compared to corresponding normal control cells (Fig. 3a). Once the NLRP3 inflammasome is activated by LPS, the expression and secretion of cleaved caspase-1 (CASP1) and IL-1β is enhanced^19^. In addition, the biological activity and function of their cleavage forms are stronger than their precursor forms. Thus, in order to verify that the expression of LXRα regulates the expression and activation of the NLRP3 inflammasome, we measured the expression levels of NLRP3, CASP1 and IL-1β in ccRCC cells with stable knockdown of LXRα (Figs. 3b, c). In accordance with expression level of NLRP3, knockdown of LXRα resulted in the up-regulation of CASP1 and IL-1β expression and enhanced their cleavage products, CASP1 (p20) and IL-1β (p15), compared with pro-CASP1 and pro-IL-1β. Meanwhile, the expression level of the NLRP3 inflammasome was dose-dependent of LPS (Fig. 3b). To prove expression of CASP1 and IL-1β was NLRP3-dependent, we transfected si-NLRP3 into ccRCC cells with stable LXRα-knockdown. In cells with stable LXRα-knockdown, si-NLRP3 transfection inhibited the up-regulation of CASP1 and IL-1β that was caused by LXRα-knockdown (Figs. 3d, e). Thus, we declared that the expression of NLRP3 in ccRCC cancer tissues was down-regulated and the expression of LXRα could dampen expression of the NLRP3 inflammasome in ccRCC cells.

### Knockdown of NLRP3 reverses the altered biological function caused by LXRα in ccRCC cells

**Fig. 4:**
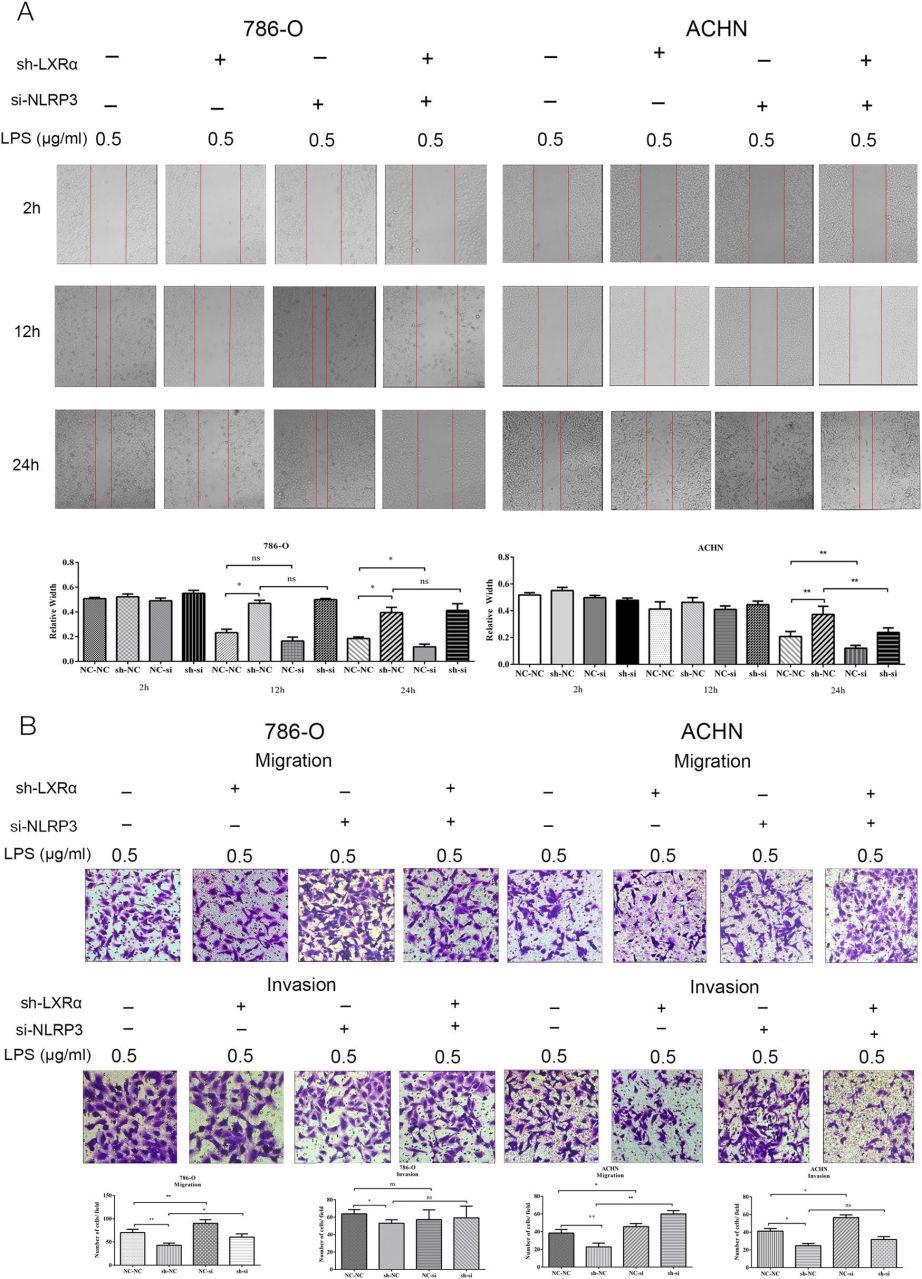
Enhanced ccRCC cell metastasis induced by LXRα is reversed by si-NLRP3. **a**, **b** Wound healing assays and transwell migration and invasion assays in ccRCC cells co-transfected with sh-LXRα and si-NLRP3. Images of wound healing assays were obtained at 2, 12, and 24 h. NC-NC, sh-NC, NC-si, and sh-si indicates NC+NC, sh-LXRα+NC, NC+si-NLRP3, and sh-LXRα+si-NLRP3, respectively. Original magnification x100 and x200 (Data are shown as mean ± SD. ****P < 0.0001; ***P < 0.001; **P < 0.01; *P < 0.05, and ns means no significant difference compared with the corresponding control)

The above results indicate that the NLRP3 inflammasome might be a possible downstream target of LXRα. To examine whether the knockdown of NLRP3 could interrupt the inhibitory effects on aggressiveness caused by LXRα knockdown in ccRCC cells, we performed rescue experiments by co-transfecting with sh-LXRα lentivirus (vs. the negative control) and si-NLRP3 (vs. the negative control) into ACHN and 786-O cells. Similar to the results shown above, transient transfection of si-NLRP3 inhibited the up-regulation of NLRP3, CASP1 and IL-1β caused by LXRα-knockdown. The wound healing assays verified that the weakening effect in cell migration induced by LXRα knockdown was effectively reversed by NLRP3 knockdown (Fig. 4a). Besides, according to the results of transwell migration and invasion assays in 786-O and ACHN that were cotransfection with sh-LXRα (vs. the negative control) and si-NLRP3 (vs. the negative control), we confirmed that knockdown of NLRP3 could reverse the inhibitory effect in ccRCC cell metastasis caused by knockdown of LXRα (Fig. 4b). It was notable that knockdown of NLRP3 could promote the cell migration and invasion in ccRCC according to our wound healing, transwell migration and invasion assays. Based on above results, we considered that the NLRP3 was tumor suppressor gene in ccRCC and the NLRP3 inflammasome is an important functional effector of LXRα in ccRCC cells for the first time.

### LXRα knockdown suppressed metastasis of ccRCC cells in vivo

**Fig. 5:**
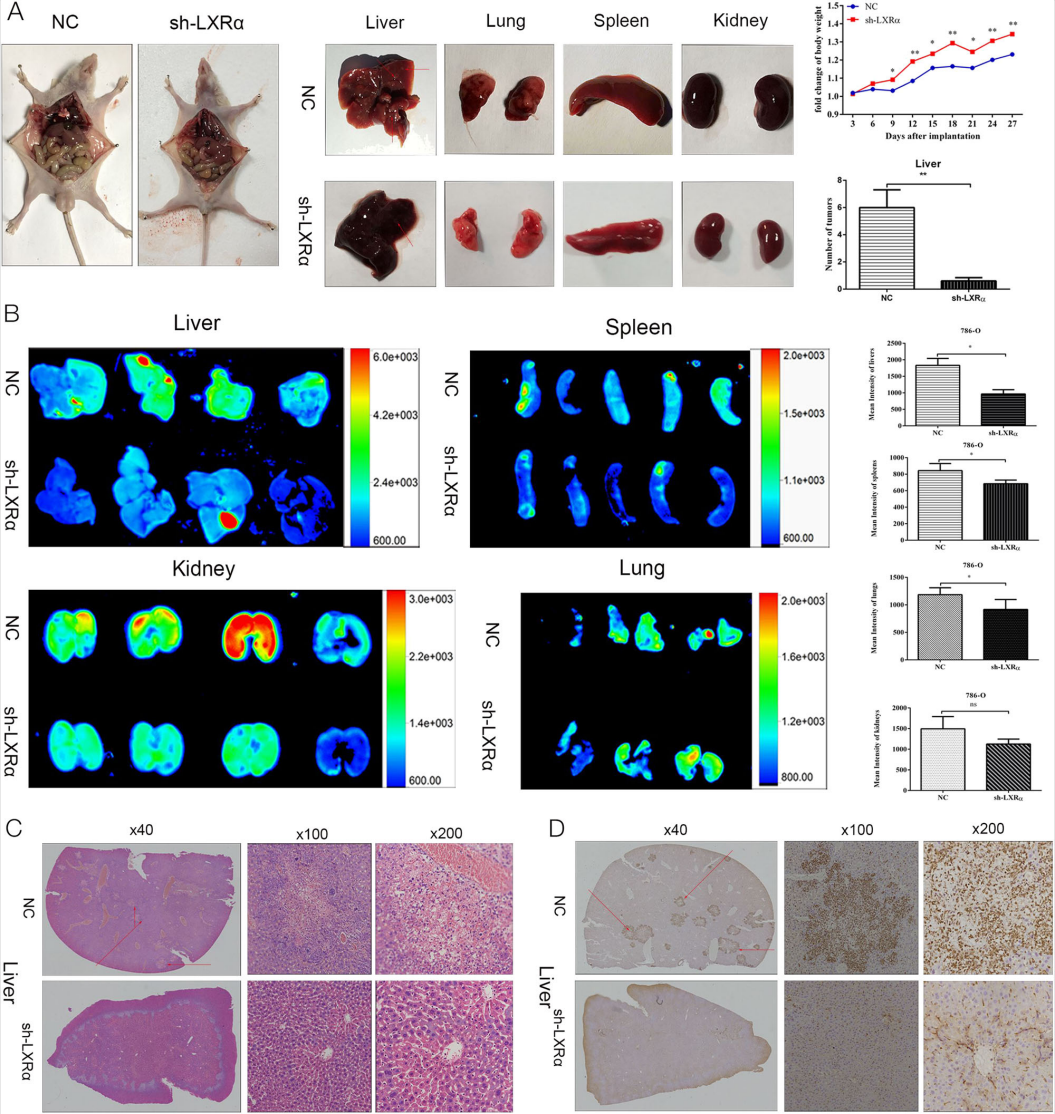
Stable knockdown of LXRα inhibits metastatic tumor properties of 786-O cells in mouse tail vein injection model. **a** Macroscopic tumor metastases in liver for 786-O tumors with stable LXRα knockdown were significantly smaller than in NC 786-O tumors. Mouse weight was maintained by stable transfection with sh-LXRα. **b** Fluorescence images and mean fluorescence intensity values of liver, spleen, kidney, and lung were analyzed in a live imaging system. **c** H&E staining in liver of tumor xenograft mouse. Original magnifications were ×40, ×100, and ×200. The metastatic tumor in liver were shown as the arrow points. **d** IHC assays of vimentin in liver of tumor xenograft mouse. The metastatic tumor in liver were revealed by dark brown. Original magnifications were ×40, ×100, and ×200. (Data are shown as mean ± SD. ****P < 0.0001; ***P < 0.001; **P < 0.01; *P < 0.05, and ns means no significant difference compared with the corresponding control)

**Fig. 6:**
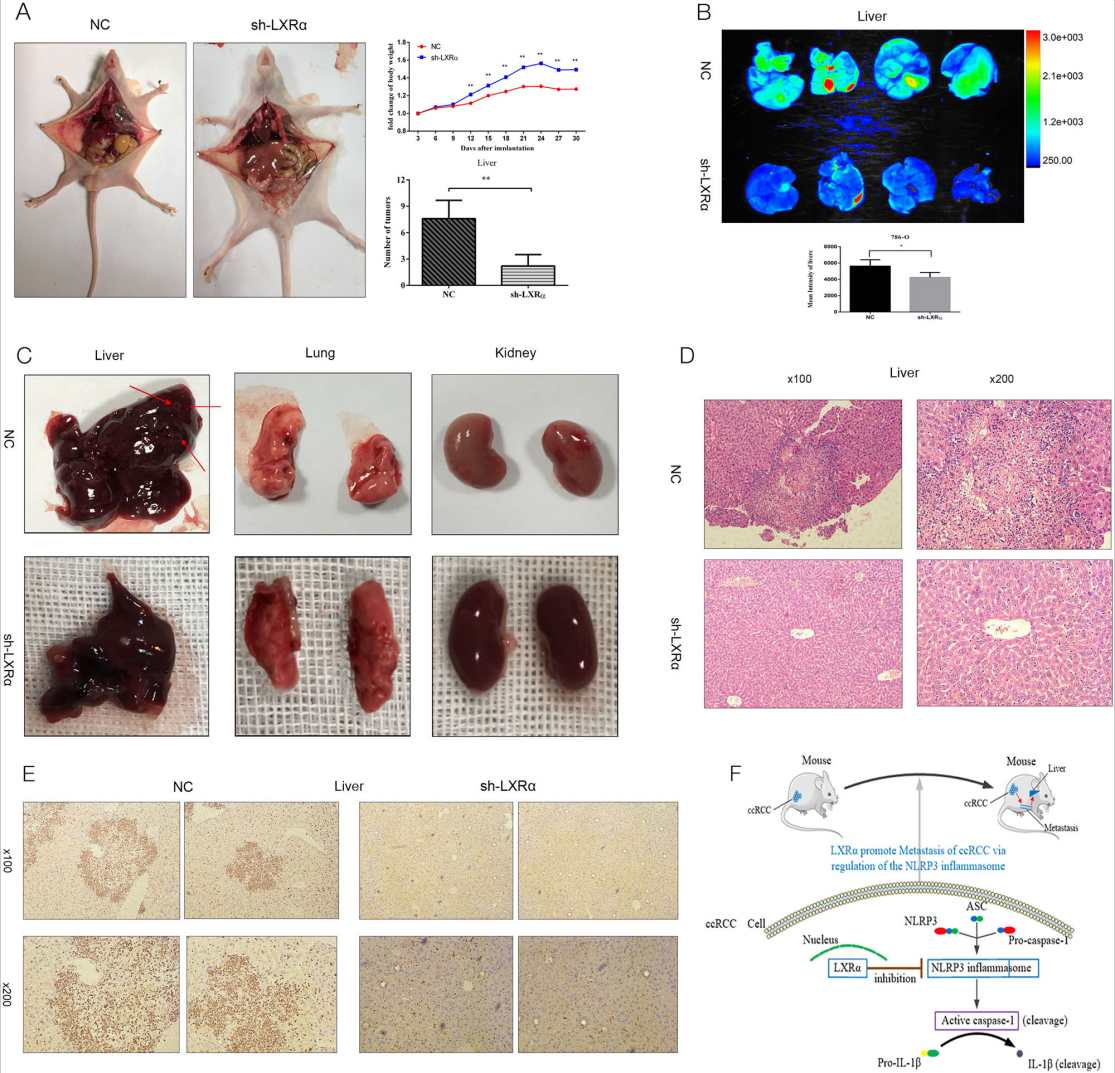
Stable knockdown of LXRα inhibits the ccRCC metastasis in mouse intrasplenical injection model. **a** The numbers of macroscopic tumor metastases in liver in NC group were more than that in sh-LXRα group. Mouse weight was measured and the fold change of body weight were calculated in NC and sh-LXRα group. **b** Fluorescence images and mean fluorescence intensity values of liver were analyzed in a live imaging system. **c** The metastatic tumor in liver were shown as the arrow points. **d** H&E staining of liver were performed. Original magnifications were ×100, and ×200. **e** IHC assays of vimentin in liver of tumor xenograft mouse. The metastatic tumor in liver were revealed by dark brown. Original magnifications were ×100, and ×200. **f** Schematic representation of elevated expression of LXRα could promote the migration and invasion of ccRCC cells via regulating the NLRP3 inflammasome (Data are shown as mean ± SD. ****P < 0.0001; ***P < 0.001; **P < 0.01; *P < 0.05, and ns means no significant difference compared with the corresponding control)

To explore the roles of LXRα in ccRCC in vivo, 786-O cells with stable knockdown of LXRα versus normal control (NC) were injected into the tail vein of nude mice. Mice were euthanized 28 days after injection, and the liver, lung, spleen and kidney were isolated and analyzed. After injection of 786-O cells, the fold change of mouse body weight in the LXRα knockdown group was significant higher than in the NC group, and the number of macroscopic metastases on the surface of the liver in the NC group was much more than that in the LXRα knockdown group (Fig. 5a). Mean fluorescence intensity values for the liver, spleen and lung in the NC group were obviously higher than the values in corresponding organs of LXRα knockdown-injected mice based on analysis of GFP expression using the automated fluorescence imaging system (Fig. 5b). The data of mean fluorescence intensity values of the organs isolated from the tumor xenograft mice could demonstrate the tumor metastasis in the mice indirectly^20^. However, the sum fluorescence intensity values for liver, spleen, lung and kidney indicated no significant difference between the sh-LXRα and NC groups (Fig. S3). H&E staining of the organs was performed, and the results demonstrated that tumor metastases were primarily distributed in the liver (Fig. 5c) (Fig. S4). Vimentin is a useful immunohistochemical marker in ccRCC that can be used to assist in distinguishing ccRCC metastatic tumors^21,22^. IHC of vimentin in this study also revealed that metastatic neoplasm numbers in the liver of LXRα knockdown groups were decreased compared to NC groups (Fig. 5d). In order to further confirm the results, liver metastases mouse model were established through intrasplenical injection. Similar to tail vein injection model, the degree of liver metastases in NC group was higher than that in sh-LXRα group according to the results of mouse weight, macroscopic metastases on the surface of the liver and automated fluorescence imaging system (Figs. 6a–c). Meanwhile, the H&E staining and IHC stain (vimentin) of livers were performed and demonstrated the same results (Figs. 6d, e). According to our results, we demonstrated that LXRα functions as an important oncogene and could modulate the metastasis of renal cancer cells by down-regulate the NLRP3 inflammasome (Fig. 6f).

## Discussion

As a cancer characterized by high mortality and metastasis rates, nearly 35% of RCC patients who have distance metastases or local relapse after nephrectomy need further chemotherapy or radiotherapy. Unfortunately, RCC is not sensitive to the majority of current chemotherapy and radiotherapy modalities for renal cancer^4^. The management of RCC, especially advanced RCC patients, requires novel and significant findings on the molecular mechanisms regulating tumorigenesis and metastasis of RCC to explore druggable biomarkers and therapeutic targets. According to the studies, several biomarkers including CAIX, SAA and CXCR4 have been proved to be prognostic biomarkers which are related to stage and aggressiveness of tumors^23–25^. However, the majority of these biomarkers are related to only some of the pathological features, diagnosis and prognosis. Thus, reliable biomarkers for diagnosis and prognostic assessment and therapeutic targets in RCC are urgently needed.

As we mentioned before, LXRs have been shown to serve as important regulators in several cancers, including breast cancer, prostate cancer. Activation of LXRs could inhibit the proliferation and invasion of prostate cancer via the beta-catenin pathway^26^. GW3965, a LXR agonist, dampen the cell proliferation in human T-cell acute lymphoblastic leukemia cells^27^. In lung cancer, LXRα is demonstrated to be an independent prognostic biomarker indicating a better survival^28^. Although the LXR agonists have been researched as anti-cancer drugs, SR9243 which is a LXR inverse agonist could inhibit the transcriptional activity of LXRs is proved to disrupt cancer cell growth^29^. The role of LXRs in RCC has not been reported up to now. According to our bioinformatics analysis results, LXRα or LXRβ expression is elevated in ccRCC indicated a poor prognosis. However, considering the degree of expression difference of LXRα or LXRβ in ccRCC cancer tissues and normal kidney tissues as well as the tissue pattern expression difference of LXRα and LXRβ, we decided to focus on the expression and biological function of LXRα in ccRCC for the remainder of the study. Intriguingly, our results demonstrate that LXRα might function as an oncogene that could serve as a novel diagnostic and prognostic biomarker in ccRCC for the first time. Additionally, bioinformatics analysis of LXRβ in ccRCC as well as qRT-PCR results of LXRβ in ccRCC indicated that LXRβ might also be an oncogene in ccRCC. The differences in expression patterns between LXRα and LXRβ and their biological function in ccRCC will be a part of our future research efforts.

According to the studies, a vital role of LXRs is modulating effects of inflammatory and immune responses via transrepression, influencing macrophage polarization and regulating fatty acid synthesis^30–32^. Most reports have focused on the regulatory effects of LXRs on inflammatory or immune responses in the tumor microenvironment or immune cells, including macrophages, regulatory T cells and monocytes^10,33^. The regulation by LXRs of tumorigenesis or metastasis in cancer via directly affecting inflammation has been rarely reported prior to this study. The inflammasome, a novel inflammatory response complex, has been a hot area for research in recent years. Based on studies revealing a correlation between LXRs and inflammation induced by LPS, we hypothesized that the NLRP3 inflammasome might be a potential target of LXRα in RCC^34^. Although increasing studies addressing the roles of the inflammasome in the development of cancers have been performed over the past few years, the function of the inflammasome in cancer is still uncertain. Several studies indicate that activation of inflammasomes is a protective factor against tumorigenesis, while other reports reach the opposing conclusion^35,36^. The role of the inflammasome in renal cancer has not been reported yet. In our study, we demonstrated that expression levels of NLRP3 in ccRCC cancer tissue were significantly lower than in normal kidney tissue. In addition, we revealed that the NLRP3 inflammasome might function as a tumor suppressor in ccRCC according to the wound healing, transwell migration and invasion assays. Furthermore, the activation of inflammasome could impact the tumor microenvironment, the anti-tumor immune responses and angiogenesis via releasing of the inflammatory factors and chemokines^37,38^. In our study, we found that the function of LXRα that was performed through the NLRP3 inflammasome was mainly focus on the metastasis in ccRCC. Thus, we conjecture that the released cytokines from the cells with active infammasome could influence the metastasis of ccRCC cells through regulation of tumor immunity or angiogenesis in ccRCC. Exploring the downstream pathways of the NLRP3 inflammasome would be another research orientation for us in future.

## Conclusions

According to our study, we found that LXRα was up-regulated and related with a poor prognosis in ccRCC. Besides, LXRα could promote the tumor metastasis by down-regulating the NLRP3 inflammasome in ccRCC. The expression of NLRP3 inflammasome was up-regulated in renal cell carcinoma compared with the normal kidney tissue. The NLRP3 inflammasome might be a tumor suppressor in RCC. According to our research, we considered that the LXRα and NLRP3 inflammasome had the possibility to be novel therapeutic targets in renal cell cancer.

## Materials and methods

### Bioinformatics analysis

Using the TCGA database, we obtained the standardized expression levels of LXR mRNA in renal cancer and their correlation to clinical features (age, gender, G stage, TNM stage, overall survival (OS), etc.). Additionally, we used the Oncomine database, including the Gumz Renal dataset, Jones Renal dataset, Yusenko Renal dataset, Lenburg Renal dataset and Beroukhim Renal dataset to assess LXR mRNA data.

### Human ccRCC clinical specimens

A total of 60 ccRCC patient samples were obtained from Wuhan Union Hospital between 2014 and 2017. The samples included both cancer tissue and adjacent normal tissue (1 cm away from the margin of the tumor tissues). We used these tissue samples to perform western blotting, quantitative real-time PCR (qRT-PCR) and IHC. All patients included in the study had no history of adjuvant therapy (chemotherapy or radiotherapy), and informed consent was obtained prior to surgery. The use of these samples was ratified by the Ethics Committee of Human Research of Huazhong University of Science and Technology (HUST).

### Cell culture, lentivirus infection, and transient transfection assay

ccRCC cell lines, including 786-O, ACHN, A498, OSRC-2, Caki-1 and a proximal tubular cell (PTC) line, HK-2, which is derived from normal kidney, were purchased from the American Type Culture Collection (ATCC) in the USA. High-glucose DMEM containing 10% FBS and 1% streptomycin-penicillin (Google Biotechnology, Wuhan, China) was used for cell culture, and cells were maintained in a 37 ℃ and 5% CO2 culture environment. Lentivirus containing pLent-LXRα-GFP-Puro or empty vector (Vector) pLent-GFP-Puro (Vigene Biology, Shandong, China) was used to infect ACHN or A498 cells with enhanced infection solution (EIS) (Vigene Biology, Shandong, China) according to the manufacturer’s protocol. Similarly, pLent-GFP-sh-LXRα-Puro lentivirus or its negative control (NC) pLent-GFP-Puro lentivirus (Vigene Biology, Shandong, China) was used to infect 786-O or ACHN cells. Short-interfering RNA (siRNA) against NLRP3 (si-NLRP3) and its negative control (si-NC) were synthesized by RiboBio (RiboBio, Guangzhou, China) and transfected into ccRCC cells using Lipofectamine 2000 reagents (Invitrogen) according to the manufacturer’s instructions.

### Inflammasome activation

After being stimulated by lipopolysaccharide (LPS) (0.5 µg/ml) for 4 h in DMEM complete medium, cells were incubated with ATP (5 mM) for 1 h in DMEM complete medium to active the intracellular inflammasome. Afterward, cells or cell supernatants were collected for molecular biology experiments.

### Quantitative reverse trancription-PCR

Total RNA was extracted from ccRCC tissues samples using Trizol Reagent BD (Invitrogen, Carlsbad, CA), and the Nanodrop 2000c spectrophotometer (Thermo Scientific) was used to determine the concentration and purity of RNA after extraction. Then, the RevertAid First Strand cDNA Synthesis Kit (Thermo Scientific, Massachusetts, USA) and reverse transcript primers (RiboBio Biology, Guangzhou, China) were used for reverse transcription of RNA. Quantitative real-time PCR (qRT-PCR) was performed with SYBR Green qPCR Mix (Invitrogen, Carlsbad, CA) using synthesized primers from TSINGKE (TSINGKE, Beijing, China). The Roche Light Cycler system (Roche, Mannheim, Germany) was used for qRT-PCR assays. The primer sequences are as follows:

GAPDH forward: 5′-CCTTCATTGACCTCAACTACA-3′;

GAPDH reverse: 5′-GCTCCTGGAAGATGGTGAT-3′;

LXRα forward: 5′-TTGCCTTGCTCATTGCT-3′;

LXRα reverse: 5′-CATCCGTGGGAACATCA-3′;

LXRβ forward: 5′-CTGCTCATCGCCATCAA-3′;

LXRβ reverse: 5′-CAGGCTCACCAGCTTCAT-3′.

### Western blotting

Briefly, protein from tissues and cells was isolated using RIPA buffer (Beyotime Institute of Biotechnology), and protein concentrations were measured with a BCA kit (Roche Molecular Biochemicals, Mannheim, Germany). Equal amounts of protein were subjected to 10% SDS-PAGE and transferred to PVDF membranes (Millipore, Bedford, USA). PVDF membranes were then immersed in 5% nonfat dried-milk for membrane blocking and incubated with primary antibodies against LXRα, NLRP3 (Sanying, Wuhan, China), caspase-1, IL-1β (Sanying, Wuhan, China), α-Tubulin (Cell Signaling Technology), β-Actin, GAPDH (Santa Cruz Biotechnology)overnight at 4 ℃. Next, blots were stained with appropriate secondary antibodies, goat anti-mouse or goat anti-rabbit (Santa Cruz Biotechnology) for 1.5–2 h at room temperature. Finally, bands were visualized with the ChemiDoc-XRS+ system (Bio-Rad, USA).

### Enzyme linked immunosorbent assay (ELISA) analysis

Cells were counted and plated into 6-well tissue culture plates in equal numbers to achieve 90% confluence. IL-1β ELISA kit (Sanying, Wuhan, China) was used to measure the concentration of IL-1β in the cellular supernatant according to the manufacturer’s protocol.

### Immunohistochemistry (IHC) and hematoxylin-eosin (H&E) staining

As previously described, tissue samples collected from ccRCC patients and tumor xenograft mice were made into paraffin blocks using the following steps: formalin fixation, dehydration and paraffin embedding. Then, IHC was performed using antibodies against LXRα (Sanying, Wuhan, China) and vimentin (Abclonal, Wuhan, China). A Hematoxylin and Eosin Staining Kit (Beyotime) was used for H&E staining according the manufacturer’s protocol.

### Wound healing, transwell migration, and invasion assays

After counting, cells were plated in equal numbers in 6-well culture plates. Vertical wounds were created with 0.1 µl pipette tips, cell confluence was approximately 95%, and the images were acquired at 2, 12, and 24 h. According to the manufacturer’s protocol, 24-well transwell chambers (Corning) were used for transwell migration and invasion assays. A total of 1x10^4^ cells were added into the upper chamber in 200 µl of serum-free medium for the migration assay, while 2 x 10^4^ cells were seeded into the top chamber, which was pre-coated with Matrigel (BD, Franklin Lakes, USA) for the invasion assay. Complete medium with 10% FBS was added to the bottom chamber to serve as a chemoattractant to stimulate migration or invasion of cells. After 24 h of incubation, cells on the upper surface of the chamber membrane were gently scraped with cotton swabs, and transmigrated cells were fixed with 100% methanol and stained with 0.1% crystal violet. Positively stained cells were photographed and counted in five random fields.

### Apoptosis measurement and cell cycle assays

Apoptosis assays were performed with the Annexin V-FITC/propidium iodide (PI) apoptosis detection kit from KeyGen Biotech according to manufacturer’s instructions. FACS (BD Biosciences) and ModFit LT software were used to detect and analyze the percentage of apoptotic cells. After being starved and synchronized in serum-free medium for 24 h, cells were collected and fixed in 75% ethanol overnight. Cells were subsequently incubated with PI and RNase A for 30 min at 37 ℃ in the dark. The percentage of cells in each cell cycle (G0/G1, S, G2/M stage) was also detected and analyzed using FACS (BD Biosciences) and ModFit LT software.

### Tumor xenograft model

786-O cells stably transfected with sh-LXRα lentivirus or negative control lentivirus were injected into 3-4-week-old BALB/C nude mice (HFK Bio-technology, Beijing, China) by tail vein injection and intrasplenical injection. Each group contained five mice. Before injection, the inflammasome in 786-O cells (NC and sh-LXRα group) were actived through the methods mention above. The mice were weighed every three days after injection. In addition, a live imaging system was used for observation of metastatic tumor lesions. Organs excised from mice were paraffin-embedded for IHC and H&E staining. IHC assays of organs were performed using the antibody against vimentin (Abclonal, Wuhan, China) as described above. The animal experiments were authorized by the Animal Ethics Committee of HUST.

### Statistical analysis

In this study, Graphpad Prism 6.0 (GraphPad Software, San Diego, California, USA) and SPSS 22.0 (IBM, New York, NY) statistical software were used. T-test was used for analysis of the significant differences in LXRs mRNA expression in normal kidney tissue and matched renal cancer tissue. Mann–Whitney test, Pearson's Chi-square test and a receiver operator characteristic curve were used for analysis of LXR expression in ccRCC subgroups, correlations between LXR expression and clinicopathological features of ccRCC, and the diagnostic values of LXR in ccRCC patients. Relationships between LXR mRNA expression and overall survival (OS) rate or disease-free survival rate were analyzed using the log-rank test and Kaplan-Meier curves. Results of *P* < 0.05 were considered significant.

## Supplementary information

Supplementary Materials

## Data Availability

You could find all data that is used for evaluating the conclusions in the paper and the Supplementary materials. Other related data of this study could be requested from the authors.
