# Supplementary material for: LXRα promotes cell metastasis by regulating the NLRP3 inflammasome in renal cell carcinoma
Source: Cell Death Dis. 2019 Feb 15;10(3):159. doi: 10.1038/s41419-019-1345-3 (PMC6377709; doi:10.1038/s41419-019-1345-3)
Supplement: Supplementary file 1 — Supplementary Materials [file 41419_2019_1345_MOESM1_ESM.doc]

**Supplementary Materials**

**
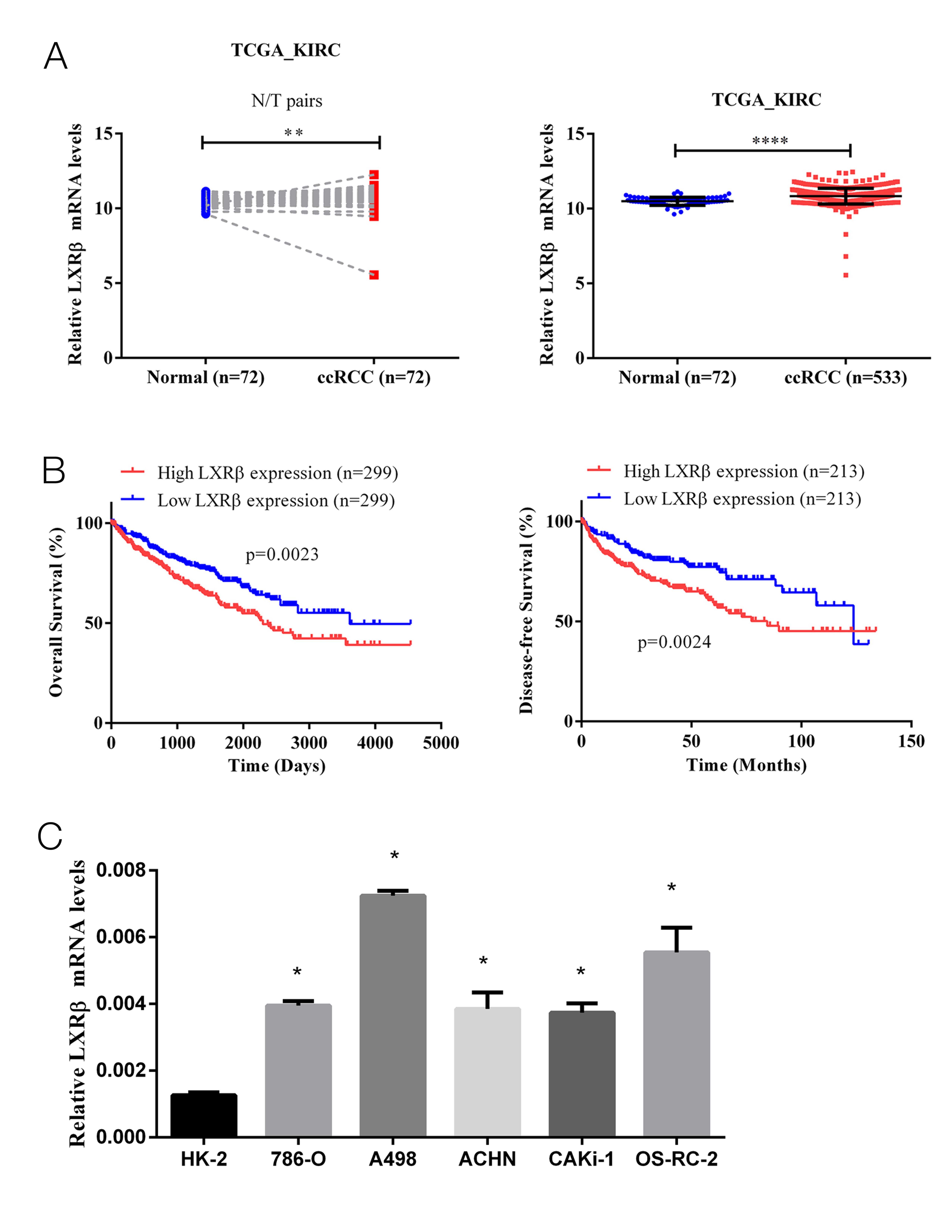
**

**Supplementary FIGURE. S1. High expression of LXRβ indicates a poor prognosis in ccRCC.** (A) mRNA levels of LXRβ in matched and unmatched ccRCC from TCGA-KIRC datasets, including 533 ccRCC tissues and 72 adjacent normal kidney tissues. (B) Data from TCGA indicated that both the OS and DFS of ccRCC patients is related to expression of LXRβ. High LXRβ expression was correlated with poor prognosis. (C) Relative LXRβ mRNA expression in ccRCC cells and HK-2 cell detected by qRT-PCR (Data are shown as mean ± SD. ****, P < 0.0001, ***, P < 0.001, **, P < 0.01, *, P < 0.05, and ns means no significant difference compared with the corresponding control).

**
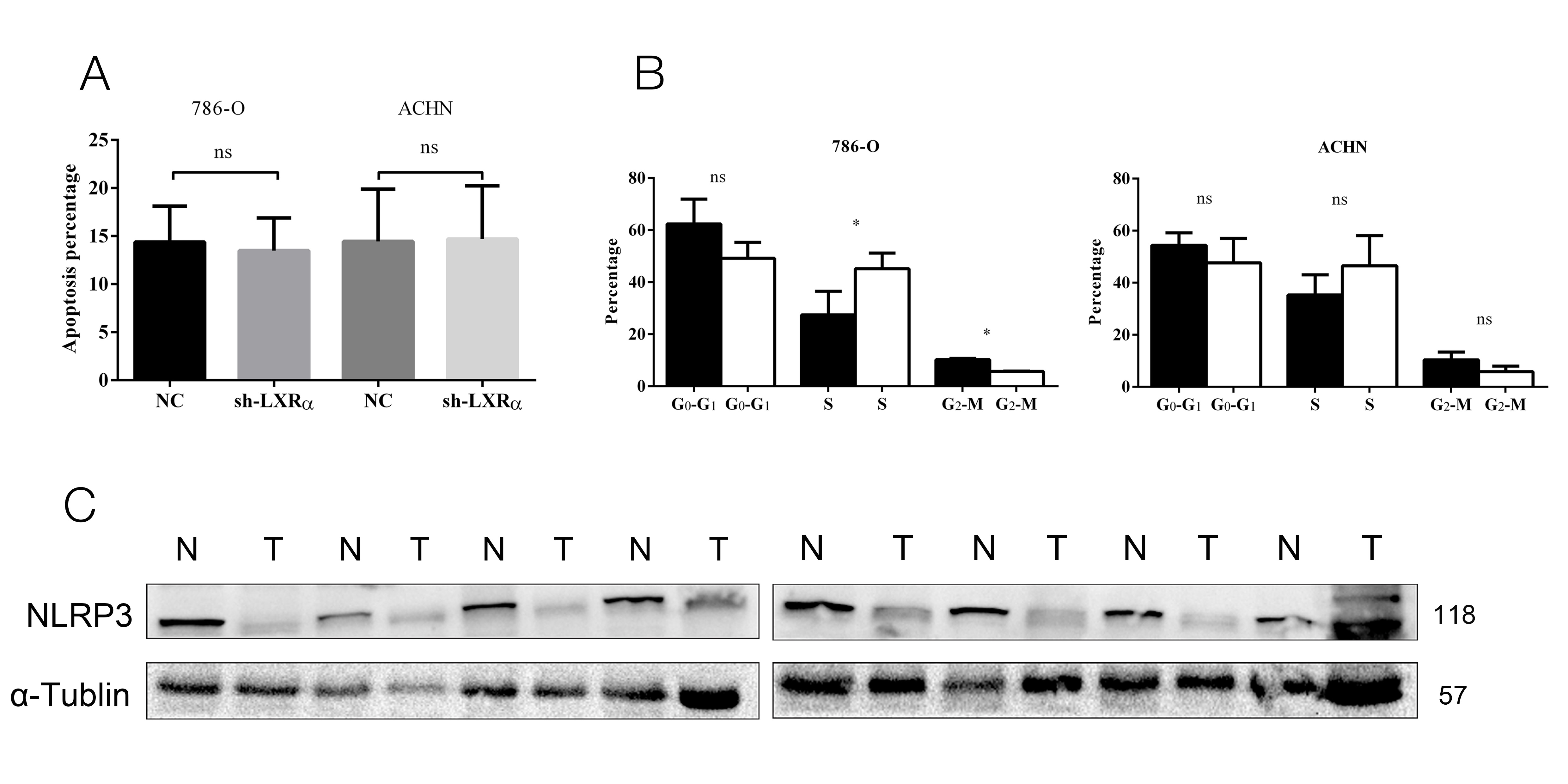
**

**Supplementary FIGURE. S2. sh-LXRα had no significant influence on apoptosis or cell cycle and the expression level of NLRP3 in ccRCC cancer tissues was down-regulated** (A) Apoptosis levels of 786-O and ACHN cells infected with sh-LXRα or control lentiviruses were monitored. (B) Cell cycle assays of 786-O and ACHN cells with stable sh-LXRα or control lentiviruses were performed. (C) The expression level of NLRP3 in ccRCC cancer tissues was down-regulated (Data are shown as mean ± SD. ****, P < 0.0001, ***, P < 0.001, **, P < 0.01, *, P < 0.05, and ns means no significant difference compared with the corresponding control).

**
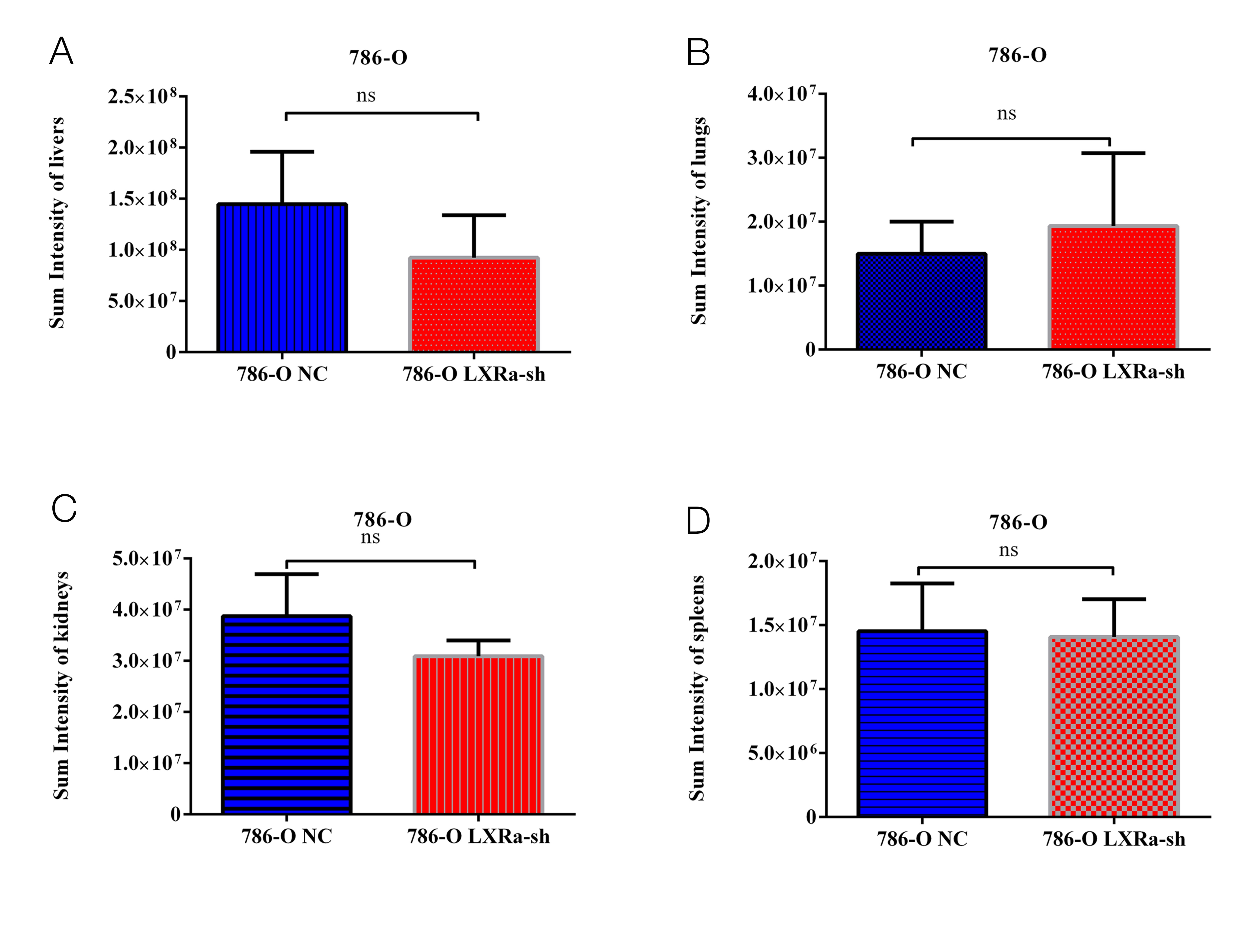
**

**Supplementary FIGURE. S3. Fluorescence intensity values of excised organs.** Fluorescence intensity values for liver (A), lung (B), kidney (C) and spleen (D) were not significantly different between NC and stable sh-LXRα groups. (Data are shown as mean ± SD. ****, P < 0.0001, ***, P < 0.001, **, P < 0.01, *, P < 0.05, and ns means no significant difference compared with the corresponding control).


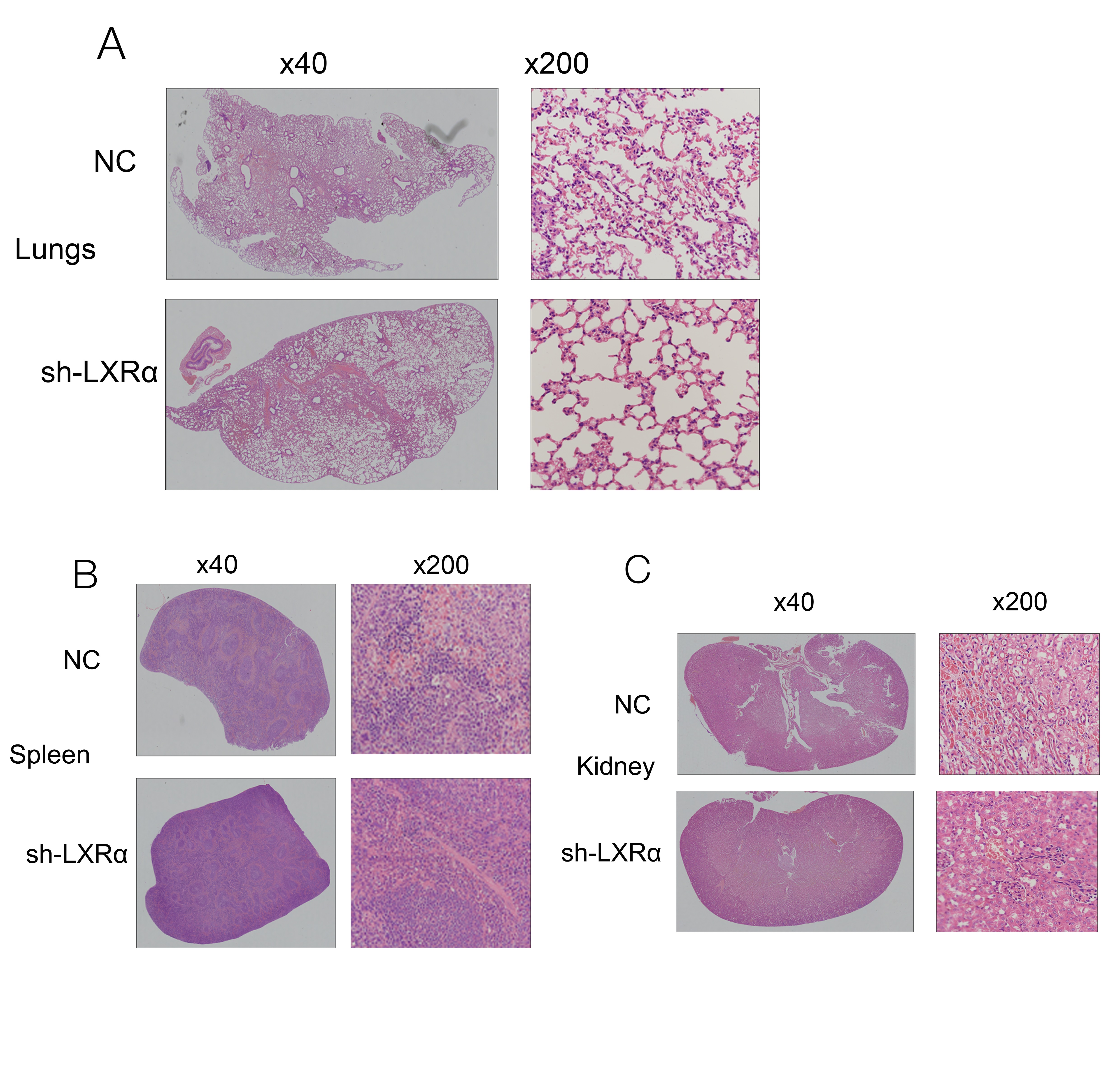


**Supplementary FIGURE. S4. H&E staining of lung, spleen, kidney tissue of tail vein injection models.** Images of H&E staining for excised lung (A), spleen (B) and kidney (C) were taken at original magnifications of × 40 and × 200.


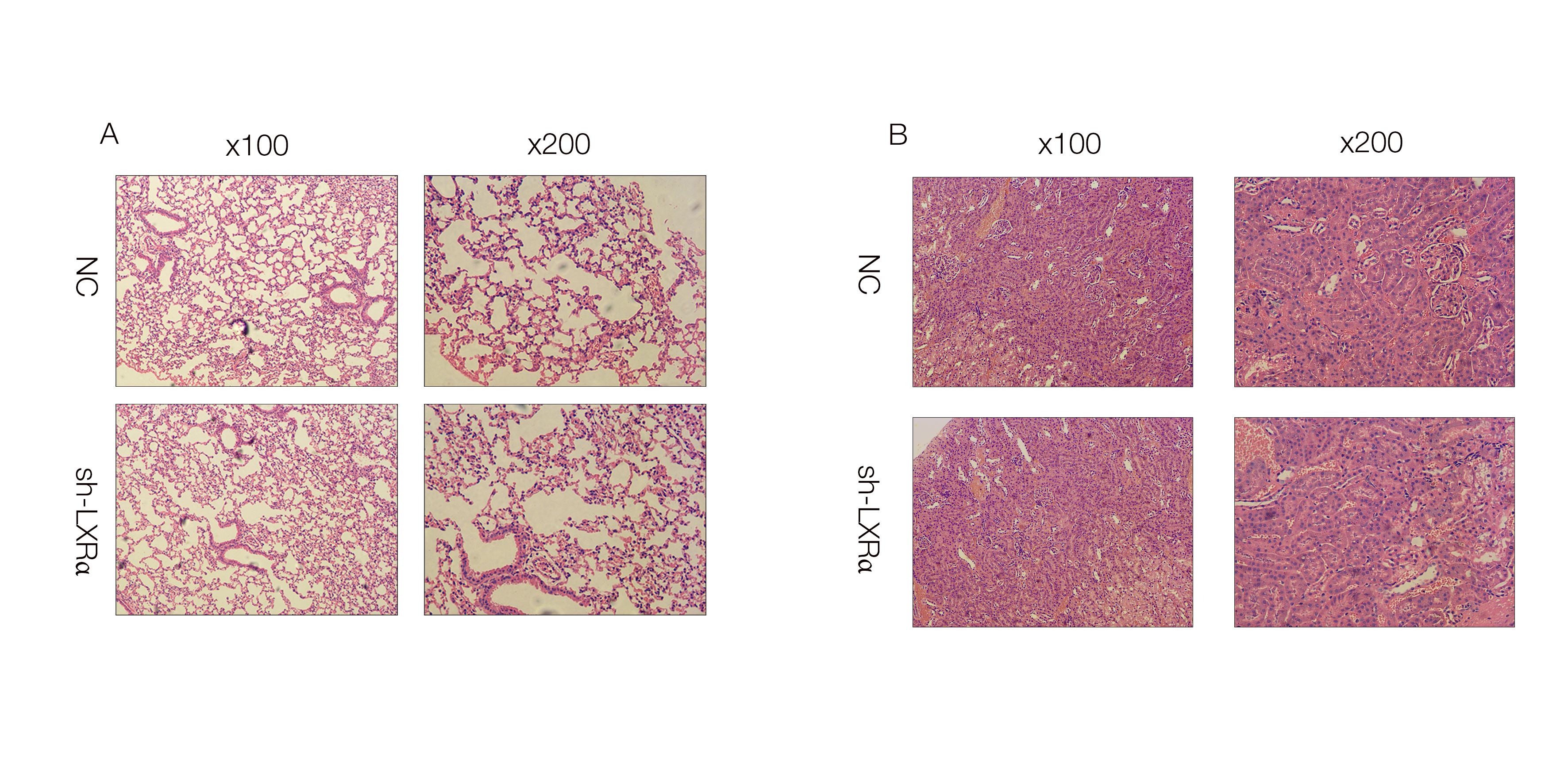


**Supplementary FIGURE. S5. H&E staining of lung and kidney tissues of mouse intrasplenical injection model.** Images of H&E staining for excised lung (A) and kidney (B) were taken at original magnifications of × 100 and × 200.
